# Epigenetically Altered T Cells Contribute to Lupus Flares

**DOI:** 10.3390/cells8020127

**Published:** 2019-02-05

**Authors:** Bruce Richardson

**Affiliations:** Department of Internal Medicine, University of Michigan Medical School, Ann Arbor, MI 48103-2200, USA; brichard@umich.edu

**Keywords:** lupus, environment, epigenetics, DNA methylation

## Abstract

Lupus flares when genetically predisposed people encounter exogenous agents such as infections and sun exposure and drugs such as procainamide and hydralazine, but the mechanisms by which these agents trigger the flares has been unclear. Current evidence indicates that procainamide and hydralazine, as well as inflammation caused by the environmental agents, can cause overexpression of genes normally silenced by DNA methylation in CD4^+^ T cells, converting them into autoreactive, proinflammatory cytotoxic cells that are sufficient to cause lupus in mice, and similar cells are found in patients with active lupus. More recent studies demonstrate that these cells comprise a distinct CD4^+^ T cell subset, making it a therapeutic target for the treatment of lupus flares. Transcriptional analyses of this subset reveal proteins uniquely expressed by this subset, which may serve as therapeutic to deplete these cells, treating lupus flares.

## 1. Etiopathogenesis of Systemic Lupus Erythematosus (SLE)

Human SLE is a systemic autoimmune disease primarily affecting women, characterized by antibody formation to autoantigens, causing immune complex generation and deposition in tissues such as the kidney and blood vessels. Family studies indicate that multiple genetic loci are involved in determining SLE susceptibility [1]. However, incomplete concordance in identical twins and the chronic relapsing course of the disease indicate that additional factors from the environment are also required for the disease to develop and flare [2,3]. Exogenous agents that trigger lupus flares include UV light exposure and infections, which cause inflammation with oxidative stress, as well as drugs such as procainamide and hydralazine, which cause lupus-like autoimmunity in genetically predisposed people [4]. The mechanisms by which these agents trigger lupus flares are incompletely understood. However, altered DNA methylation patterns in CD4^+^ T lymphocytes are caused by the environment–host interactions, and the epigenetically altered T cells are sufficient to cause lupus-like autoimmunity in animal models [5]. Insights into the mechanisms by which environmental agents alter T cell DNA methylation and cellular functions derive from studies on the role of DNA methylation in regulating gene expression in mature T cells. These led first to insights into mechanisms causing drug-induced lupus and later into the pathogenesis of idiopathic lupus.

## 2. DNA Methylation and T Cell Gene Expression

DNA methylation refers to the methylation of dC bases located in CpG pairs to form deoxymethylcytosine (d^m^C) and is a transcriptionally repressive modification. DNA methylation patterns are established during differentiation by the de novo DNA methyltransferases Dnmt3a and Dnmt3b, and serve to suppress expression of genes that would be inappropriate or detrimental to the function of any given cell, but which might be activated by transcription factors expressed by the cell. Methylcytosine binding proteins such as MBD1, MBD2, and MeCP2 then bind the methylated bases and attract chromatin inactivation complexes that promote condensation of the DNA into a transcriptionally repressive structure [6]. The patterns are then replicated by DNA methyltransferase 1 (Dnmt1) each time the mature cell divides. Dnmt1 binds the replication fork and “reads” CG pairs. Where the parent strand is methylated, Dnmt1 catalyzes the transfer of methyl groups from S-adenosylmethionine (SAM) to the corresponding dC base in the daughter strand, thereby copying the methylation patterns from the parent to the daughter strand. Importantly, environmental agents that prevent upregulation of Dnmt1 during mitosis or inhibit its activity will prevent methylation of the daughter strand, permitting inappropriate expression of normally silenced genes in the daughter cells, and the inappropriate methylation patterns may then be replicated through subsequent rounds of cell division. 

T lymphocytes are particularly sensitive to DNA methylation inhibition [7]. T cell DNA methylation patterns are established as the cells differentiate in the thymus, and serve to suppress the expression of genes that would be inappropriate for the function of any given T cell subset, but for which the cells may express transcription factors that would activate expression of the gene. Initial studies demonstrated that inhibiting the replication of methylation patterns during mitosis in cloned or polyclonal CD4^+^ T cells with the DNA methyltransferase inhibitor 5-azacytidine (5-azaC) alters gene expression, converting normal antigen-specific cells into autoreactive cells, which can respond to autologous or syngeneic macrophages lacking the appropriate antigen peptide in the binding site of the class II MHC molecule, thus becoming autoreactive [5]. The autoreactivity is due to overexpression of *ITGAL* (CD11a), a subunit of the adhesion molecule LFA-1 (CD11a/CD18), due to demethylation of the *ITGAL* promoter, and LFA-1 overexpression by transfection causes a similar autoreactivity in antigen-specific T cells [8]. The epigenetically altered T cells also overexpress perforin, normally expressed by cytotoxic cells but not by “helper” CD4^+^ T cells [9], as well as interferon gamma [10], the B cell costimulatory molecules CD70 [11] and CD40L [12], and the killer cell immunoglobulin-like receptor (KIR) genes [13]. The human KIR locus encodes 17 genes, many of which show large variation between individuals due to the high number of allelic variants and copy number variations [13]. The KIR genes are clonally expressed by NK cells but not by T cells [14]. However, inhibiting DNA methylation in human CD4^+^ T cells activates expression of the entire KIR gene family [13]. Subsequent studies, performed after the development of multicolor flow cytometry, demonstrated that these genes are all coexpressed together on the same CD3^+^CD4^+^CD28^+^ T cell, defining a novel CD3^+^CD4^+^CD28^+^CD11a^high^CD70^+^CD40L^high^KIR^+^ subset [15]. A more recent study using genomics approaches identified 1897 genes differentially expressed by the epigenetically altered cells [16]. This study also identified 718 hypomethylated and overexpressed genes in the KIR^+^CD11a^high^ compared to autologous KIR^−^CD11a^low^ T cell subset. Bioinformatics analysis of these 718 genes revealed significant enrichment in proinflammatory gene ontologies, pathways, and gene metagroups. The most significant gene ontologies enriched in this subset point to a positive regulation of the immune response, and the most significant pathway is “graft versus host disease”, which has clinical features resembling human lupus [17]. Importantly, as noted above, the KIR proteins are clonally expressed on NK cells but not on normal T cells, while CD4^+^ T cells epigenetically altered with DNA methylation inhibitors express all the KIR genes. This suggests that antibodies to one or a limited number of KIR proteins would eliminate all the epigenetically altered T cells but only a limited number of NK cells. More recent studies demonstrate that IL-17a is regulated by histone methylation.

## 3. DNA Demethylation and T Cell Function

The effects of the changes in gene expression on T cell effector function were studied in vitro using human and murine T cells. These studies demonstrated that the experimentally demethylated, autoreactive CD4^+^ T cells are cytotoxic and induce apoptosis in autologous or syngeneic macrophages, causing release of antigenic apoptotic chromatin as well as impairing its clearance [18]. Others have reported that injecting apoptotic cells into mice, or impairing apoptotic cell clearance by genetic manipulation, is sufficient to cause anti-DNA antibodies and a lupus-like disease in mice [19], suggesting that the macrophage apoptosis mediated by the demethylated T cells releases chromatin that contributes to anti-dsDNA antibody development. This was tested using murine models. CD4^+^ murine T cells become autoreactive following treatment with DNA methylation inhibitors. When the treated cells are injected intravenously into syngeneic mice, the demethylated cells accumulate in the spleen where they can respond to and cause the macrophage apoptosis described by others [20] and provide B cell costimulatory signals that cause immunoglobulin overproduction [11,21]. The increased macrophage apoptosis, together with impaired clearance of apoptotic debris, normally done by the macrophages, results in anti-DNA antibody formation in non-lupus-prone mice [18] and anti-DNA antibodies with renal immune complex deposition in lupus-prone SJL mice [22]. Importantly, removing the recipient’s spleen before the injection prevents interactions between the epigenetically altered T cells with B cells and macrophages, preventing autoantibody and disease development [23]. 

## 4. T Cell DNA Demethylation in Drug-Induced and Idiopathic Lupus

The observation that CD4^+^ T cells treated with the DNA methylation inhibitor 5-azaC could cause a lupus-like disease suggested that drugs which cause lupus may be DNA methylation inhibitors. Procainamide, an antiarrhythmic, and hydralazine, an antihypertensive agent, both cause lupus-like autoimmunity in genetically predisposed people [24]. Initial studies demonstrated that CD4^+^ T cells also become autoreactive following treatment with these drugs [24]. Subsequent studies demonstrated that procainamide is a competitive inhibitor of DNA methyltransferase enzymatic activity [25], while hydralazine inhibits T cell ERK pathway signaling, which prevents Dnmt1 upregulation when CD4^+^ T cells entered mitosis [26]. The pathologic significance of the epigenetically altered cells was tested by treating murine CD4^+^ T cells with 5-azaC or procainamide and then injecting them into syngeneic recipients. Mice receiving the treated T cells, but not untreated T cells, developed identical lupus-like autoimmunity with anti-DNA and anti-histone antibodies and an immune complex glomerulonephritis [27].

The observation that CD4^+^ T cells epigenetically altered with DNA methylation inhibitors could cause lupus-like autoimmunity in mice suggested that patients with idiopathic lupus might have similar epigenetically altered T cells. Early studies demonstrated that T cells from patients with active, but not inactive, lupus have hypomethylated DNA [28]. Subsequent studies demonstrated low Dnmt1 levels in T cells from patients with active lupus [29]. Dnmt1 levels are normally upregulated by signals transmitted through the ERK and JNK pathways as T cells enter mitosis, and ERK pathway signaling is decreased in T cells from active lupus patients, contributing to the low Dnmt1 levels [22]. The lupus ERK pathway signaling defect was traced to PKCδ inactivation caused by nitration, a form of oxidative damage caused by superoxide (O_2_^−^) combining with nitric oxide (NO), an intracellular signaling molecule, to form peroxynitrite (ONOO^−^), which nitrates tyrosine, and the inactive PKCδ fraction in lupus T cells is nitrated [30]. Importantly, lupus flares are characterized by extensive serum protein nitration, generated during inflammatory responses [31].

**Figure 1:**
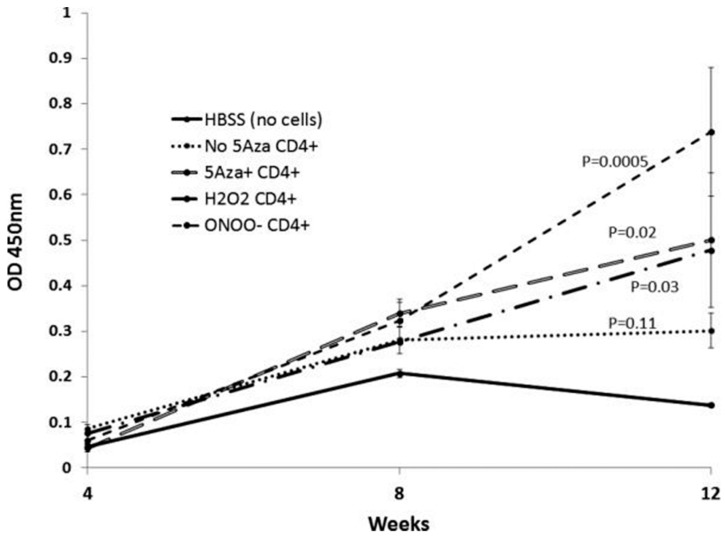
Splenocytes from SJL mice were stimulated with concanavalin A, treated as indicated, then injected into syngeneic recipients every two weeks for a total of seven injections. Anti-dsDNA antibodies were measured by enzyme-linked immunosorbent assays (ELISA) at the indicated weeks. The Y axis shows the optical density (OD) of the ELISA.

**Figure 2:**
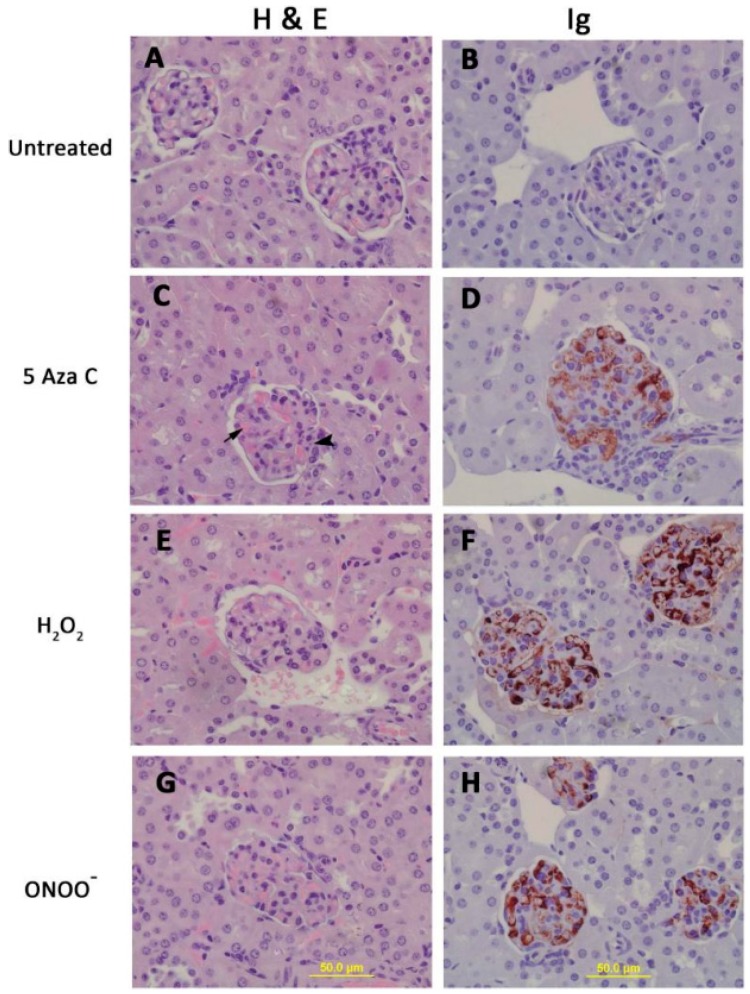
CD4^+^ T cells treated with 5-azaC, H_2_O_2_ or ONOO^−^ cause an immune complex glomerulonephritis. Kidneys from the mice shown in Figure 1 were removed for histologic analysis. Hematoxylin and eosin stained kidney sections from mice receiving untreated (**A**), 5-azaC treated (**C**), H_2_O_2_ treated (**E**) or ONOO^−^ treated (**G**) CD4^+^ T cells. Panels **B**, **C**, **F** and **H** show glomeruli from the same mice stgained for IgG deposition using immunoperoxidase staining.

The pathologic significance of T cell protein nitration was tested by treating stimulated CD4^+^ female SJL mouse T cells with H_2_O_2_, ONOO^−^, or the Dnmt1 inhibitor 5-azacytidine (5-azaC), then injecting the treated cells into syngeneic female recipients. Controls included mice receiving stimulated, untreated CD4^+^ T cells. The treated cells all overexpressed the methylation sensitive genes CD40L and KIR, and mice receiving the treated cells, but not the untreated cells, all developed anti-dsDNA antibodies (Figure 1) and glomerulonephritis (Figure 2) [32]. This indicates a mechanism by which environmental agents such as UV light and infections may trigger lupus flares.

**Figure 3:**
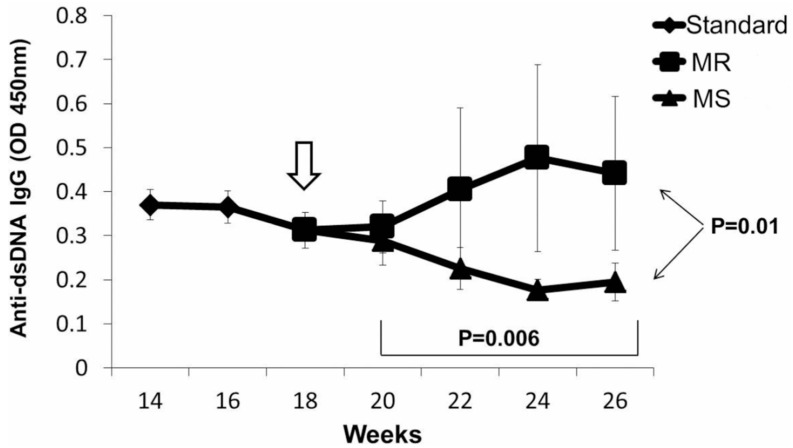
Anti-dsDNA IgG Antibody Levels Decline in DOX-Treated Transgenic Mice Fed the MS Diet. Standard diet weeks 14–18 vs. MS diet weeks 20–26, *p* = 0.006 (linear regression); Standard diet vs. MR diet *p* = 0.368 (linear regression). MS vs. MR diet, week 26, *p* = 0.01.

The replication of DNA methylation patterns during mitosis requires not only adequate levels of Dnmt1 during mitosis but also requires SAM, which donates the methyl group required to methylate the cytosines in the newly synthesized DNA strand [33]. SAM is formed from methionine, an essential amino acid, and is obtained from the diet [34]. This suggests that diet may also be an important exogenous agent affecting lupus flare severity. This was tested using in vitro and in vivo models. Later studies demonstrated that a diet deficient in methyl donors could increase anti-DNA antibody levels and severity of lupus nephritis in a murine lupus model in which lupus flares are caused by selectively decreasing Dnmt1 levels in T cells, while a diet supplemented with methyl donors decreased anti-DNA antibody levels and the severity of the nephritis (Figure 3) [35]. Others have described the importance of diet in lupus as well [36], and the microbiome can contribute to the generation of oxidative stress [37].

## 5. Summary

Together, these studies indicate that exogenous agents can induce lupus flares by decreasing CD4^+^ T cell DNA methylation levels through mechanisms including decreasing Dnmt1 activity, either by inhibiting its enzymatic activity or decreasing Dnmt1 levels through effects on signaling pathways, or by decreasing the bioavailability of dietary methyl donors. This suggests a mechanism by which antioxidants such as N-acetylcysteine may be beneficial in lupus patients and that attention to proper dietary habits or supplements may also be beneficial in the management of lupus patients.
